# Low PG I/II ratio as a marker of atrophic gastritis: Association with nutritional and metabolic status in healthy people

**DOI:** 10.1097/MD.0000000000010820

**Published:** 2018-05-18

**Authors:** Weiwei Su, Bin Zhou, Guangming Qin, Zhihao Chen, Xiaoge Geng, Xiaojun Chen, Wensheng Pan

**Affiliations:** a Department of Ultrasonography; b Department of Gastroenterology; c Department of Laboratory, Second Affiliated Hospital, School of Medicine, Zhejiang University; d Department of Gastroenterology and Endoscopy Center, Zhejiang Provincial People's Hospital, People's Hospital of Hangzhou Medical College, Hangzhou 310009, China.

**Keywords:** atrophic gastritis, metabolism, nutrition, pepsinogen

## Abstract

A low pepsinogen (PG) I/II ratio can be used to detect atrophic gastritis (AG). Recent research has found that the PG I/II ratio is associated with several nutritional and metabolic disorders. The aim of this study is to investigate the relationship between the PG I/II ratio and biochemical markers in a Chinese population.

In total, 1896 participants in a gastric cancer screening program underwent a health screening test that included assessment of serum pepsinogens. Subjects with PG I/II < 3.0 were considered as having atrophic gastritis. Associations between the PG I/II ratio and biochemical markers reflecting glucose and lipid metabolism, liver, kidney and thyroid functions were evaluated using SPSS software version 20.

The prevalence of atrophic gastritis was 5.3% and increased with age but did not differ between sexes. Albumin, ferritin, and total and direct bilirubin were significantly lower in patients with AG than in those without AG, whereas age, total bile acid, and amylase were significantly higher. Albumin, ferritin, and triglyceride correlated positively with the PG I/II ratio, while age, total bile acid, blood urea nitrogen, amylase, aspartate aminotransferase, creatine kinase, and lactate dehydrogenase correlated inversely with the PG I/II ratio. Logistic regression analysis demonstrated that age, total bile acid, total protein, and ferritin correlated independently with AG.

Low PG I/II ratio is not only a marker of atrophic gastritis but also an indicator of nutritional and metabolic status. Special attention should be paid to the metabolism of iron, protein, and bile acid in patients with a low PG I/II ratio.

## 1 Introduction

Gastric cancer is one of the most common malignant gastrointestinal tumors and has high morbidity and mortality.^[1,2]^ The gastric mucosa undergoes a stepwise process of inflammation-atrophy-metaplasia-dysplasia before developing into gastric cancer.^[3]^ Atrophic gastritis (AG), an important step in this sequence, is characterized by loss of gastric glands and can be assessed endoscopically, histologically, and serologically. Upper gastrointestinal endoscopy with biopsy is the gold standard for the diagnosis of AG, but this procedure is invasive, and tissue sampling error may occur. Serum pepsinogens (PGs), referred to as “serologic biopsy”, are noninvasive diagnostic biomarkers for AG. Pepsinogens are the inactive precursors of pepsin and can be categorized into 2 types, PG I and PG II. PG I is exclusively secreted by the chief and mucous neck cells of the corpus and fundic glands, and PG II is also secreted by the pylori glands and Brunner's glands. Approximately, 1% of the pepsinogens secreted into the gastric lumen are permeated into blood circulation and thus detected in serum. In this way, serum pepsinogen levels can be used to reflect the morphological and functional status of gastric mucosa. With atrophy of mucosal glands in the antrum and/or corpus, the levels of PG I and PGII change correspondingly, resulting in a decrease of the PG I/II ratio. It has been found that the serum PG I/II ratio is closely correlated with histologic atrophic gastritis, and PG I/II < 3.0 is considered to be the optimal cut-off value for atrophic gastritis with high sensitivity and specificity.^[4]^

Atrophic gastritis, that is, gastric mucosal atrophy, will cause hypo- or achlorhydria, intrinsic factor deficiency and ultimately bacterial overgrowth in the stomach and proximal small intestine.^[5–7]^ Therefore, absorption and metabolism of dietary nutrients, essential vitamins (like vitamin B_12_), micronutrients (like iron, calcium, and magnesium), and certain medicines (like dipyridamol, fluconazole, and thyroxin) are impaired in patients with atrophic gastritis.^[8–10]^ In recent years, pepsinogens have been found to be associated with several nutritional and metabolic disorders including obesity, dyslipidemia, hyperuricemia, hypertension, and diabetes mellitus,^[11–13]^ irrespective of *Helicobacter pylori* (Hp) infection. The PG I/II ratio is correlated with glucose, triacylglycerol, and uric acid levels^[13]^ and is valuable for predicting nephropathy in patients with hypertension^[11]^ and type 2 diabetes mellitus.^[12]^

We assumed that the PG I/II ratio, as a marker of atrophic gastritis, may be associated with nutritional and metabolic status in healthy people. However, to the best of our knowledge, there are few studies exploring the relation between the PG I/II ratio and biochemical markers reflecting nutritional and metabolic status, such as glucose, lipids, liver, kidney and thyroid function. Therefore, we conducted research to investigate the characteristics of serum pepsinogen levels and their relation to biochemical markers in a healthy Chinese population.

## 2 Methods

### 2.1 Subjects

A population-based gastric cancer screening program (the Key Project of Zhejiang Province, No. 2013C03044-5) has been conducted in Zhejiang Province since 2014 to improve the detection rate of gastric cancer by using stratified screening and follow-up in healthy people. In total, 2490 participants underwent a health screening test that included measurement of serum pepsinogens, from January 2014 to December 2014, at the Second Affiliated Hospital of Zhejiang University School of Medicine. Subjects were excluded if they had hypertension, diabetes mellitus, hyperlipidemia, hyperuricemia or a history of stomach surgery, *H pylori* eradication or medication administration of a proton pump inhibitor within 4 weeks. After the additional exclusion of subjects with incomplete data, 1896 subjects were enrolled in the cross-sectional analysis. All subjects were divided into 2 groups according to PG I/II ratio; those subjects with PG I/II < 3.0 were considered as having atrophic gastritis. All the subjects provided written consent, and the study was reviewed and approved by the institutional review board of the Second Affiliated Hospital of the Zhejiang University School of Medicine (ethical review code: Research 2014-170).

### 2.2 Laboratory data

A fasting blood specimen was obtained from each subject for measurement of levels of various biochemical markers. Biochemical analysis was performed using Beckman Coulter AU5800 (Beckman Coulter Inc., Brea, CA), and thyroid hormones, insulin, ferritin, and pepsinogen were assessed by Abbott Architect i2000SR (Abbott Diagnostic, Chicago, IL), per the manufacturer's instructions and standard operating procedures. Serum pepsinogens were measured with chemiluminescent enzyme immunoassay (Abbott ARCHITECT Pepsinogen I/II Reagent Kit, Abbott Laboratories, Chicago, IL). Aspartate aminotransferase (AST), alanine transferase, alkaline phosphatase, γ-glutamyl transpeptidase, total bilirubin (TBil), direct bilirubin (DBil), indirect bilirubin (iDBil), total bile acid (TBA), and albumin (ALB) were used for reflecting liver function; blood urea nitrogen (BUN), creatinine and uric acid were used for renal function; glucose and insulin were used for glucose metabolism; total cholesterol, triglyceride (TG), high-density lipoprotein, low-density lipoprotein, and apolipoproteinA_1_/B were used for lipid metabolism; and total/free triiodothyronine, total/free thyroxine and thyroid stimulating hormone were used for thyroid function. The reference ranges of each measured biomarker are listed in Table 1.

**Table 1:**
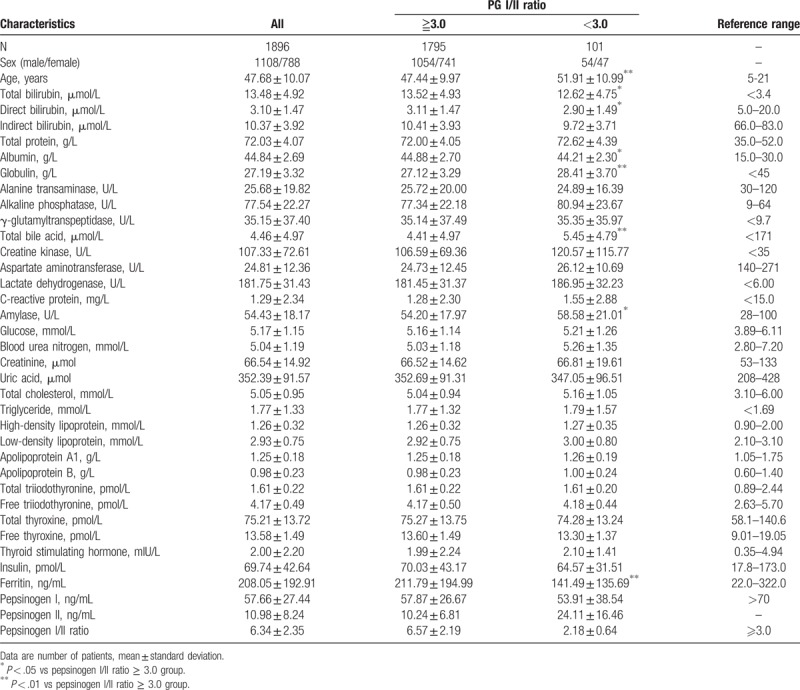
Characteristics of all subjects.

### 2.3 Statistical analysis

Categorical variables are presented as number and continuous variables are presented as the mean ± standard deviation. Skewed-distributed variables were logarithmic transformed before statistical analyses of variance, correlation and regression. Comparison between the 2 groups was examined by unpaired student's *t*-test, and association between PG I/II ratio and other variables was assessed by Pearson's correlation analysis. Logistic regression analysis was performed to explore the influence of different variables on atrophic gastritis defined as PG I/II < 3.0. *P*-value < .05 was considered statistically significant. All statistical analyses were performed using SPSS 22 software (SPSS Inc. Chicago).

## 3 Results

Atrophic gastritis, defined as PG I/II < 3.0, was found in 101 (5.3%) of the 1896 subjects, and the mean value of the PG I/II ratio was 6.34 ± 2.35 in all subjects, 6.57 ± 2.19 in the PG I/II ratio ≥ 3.0 group, and 2.18 ± 0.64 in PG I/II ratio < 3.0 group, respectively.

PG I/II ratio decreased significantly with age (correlation coefficient: −0.146, *P* = .000) but did not differ between sexes (χ^2^ = 1.086, *P* = .297).

**Figure 1:**
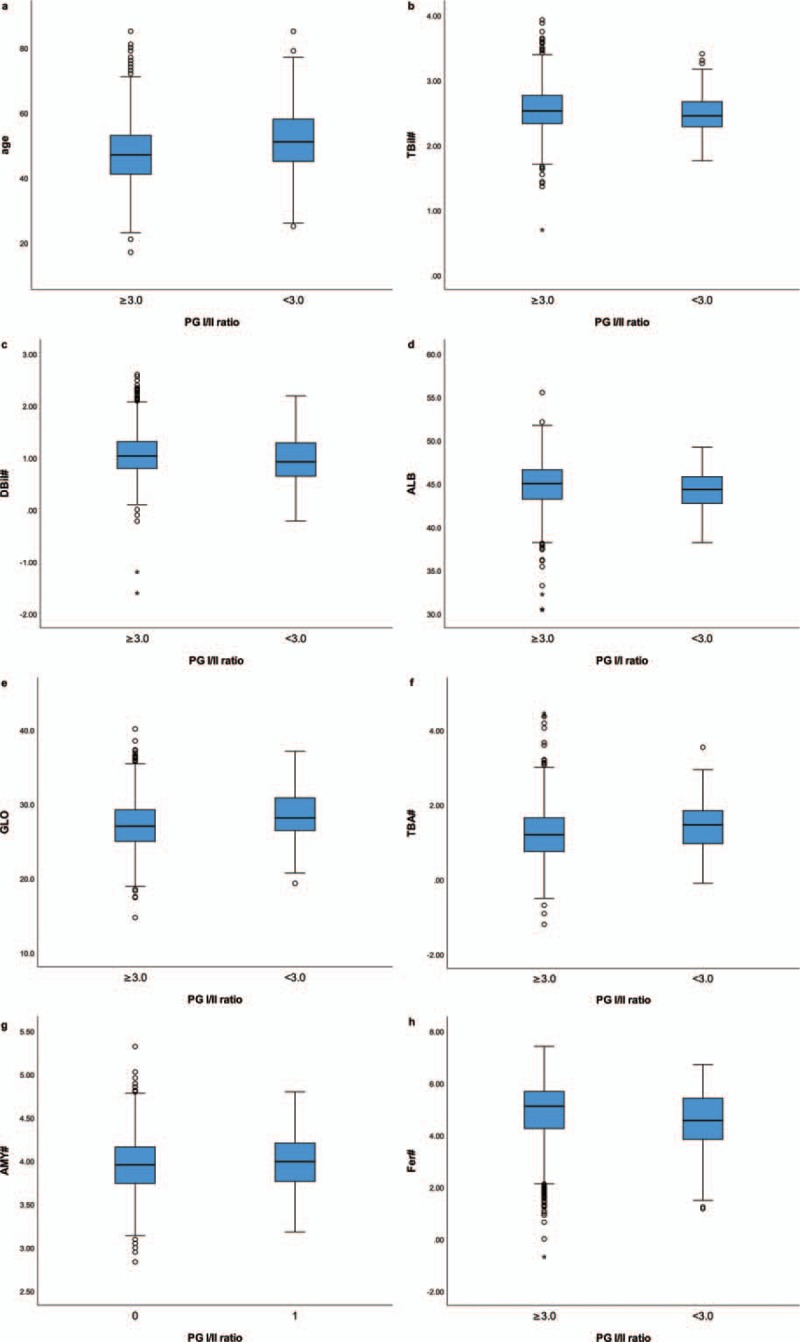
Box plots for the variables with significant difference between groups. (A) Age. (B) TBil = total bilirubin. (C) DBil = direct bilirubin. (D) ALB = albumin (E) GLO = globulin. (F) TBA = total bile acid. (G) AMY = amylase. (H) Fer = ferritin. ^#^Variables were logarithmic transformed due to skewed distribution.

Albumin, ferritin, and total and direct bilirubin were significantly lower, whereas age, total bile acid, and amylase were significantly higher in patients with AG than in those without AG. Characteristics of all the subjects are shown in Table 1. Box plots for the variables with significant difference are listed in Figure 1.

**Figure 2:**
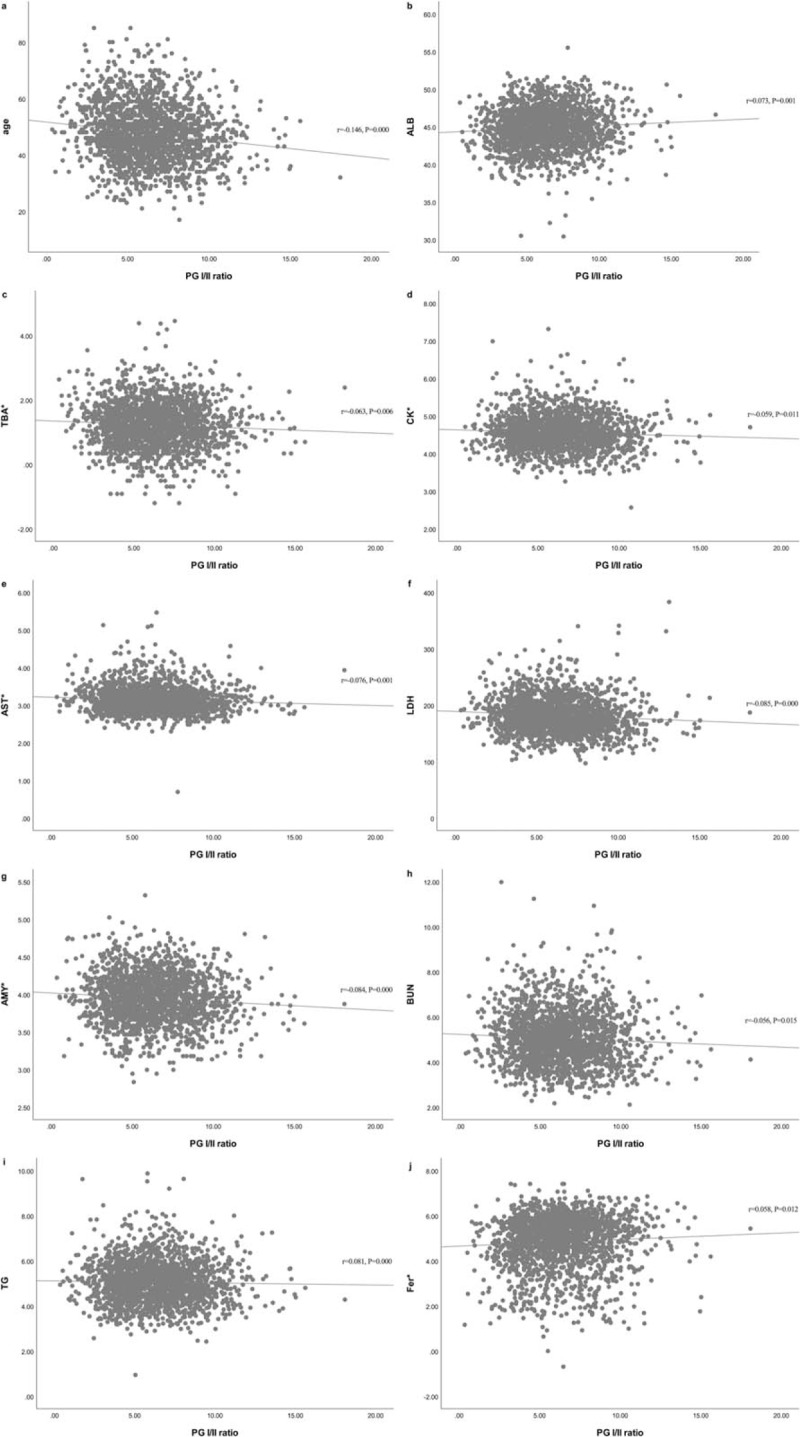
Scatter plots for correlation between the PG I/II ratio and other variables. (A) Age. (B) ALB = albumin. (C) TBA = total bile acid. (D) CK = creatine kinase. (E) AST = aspartate aminotransferase. (F) LDH = lactate dehydrogenase. (G) AMY = amylase. (H) BUN = blood urea nitrogen. (I) TG = triglyceride. (J) Fer = ferritin. ^∗^Variables were logarithmic transformed due to skewed distribution.

Albumin, ferritin, and triglyceride correlated positively with PG I/II ratio, while age, total bile acid, blood urea nitrogen, amylase, aspartate aminotransferase, creatine kinase, and lactate dehydrogenase correlated inversely with PG I/II ratio. Relationships between PG I/II ratio and other variables are shown in Table 2. Scatter plots for correlation between variables and PG I/II ratio are listed in Figure 2.

**Table 2:**
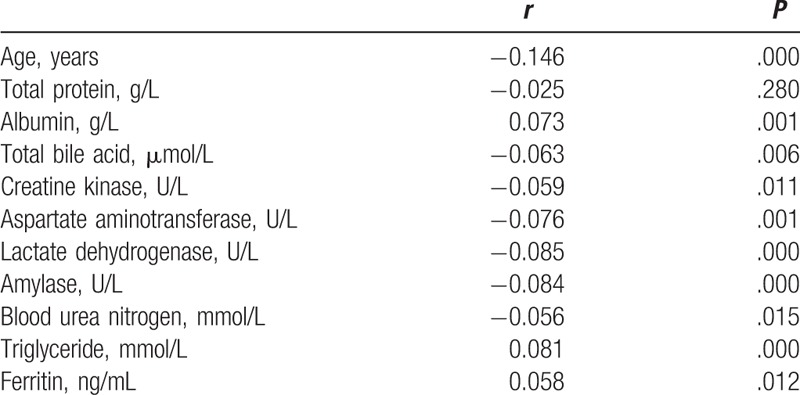
Correlation between the pepsinogen I/II ratio and other variables (only partial variables listed).

Logistic regression analysis demonstrated that age, total bile acid, total protein, and ferritin correlated independently with AG (Table 3).

**Table 3:**
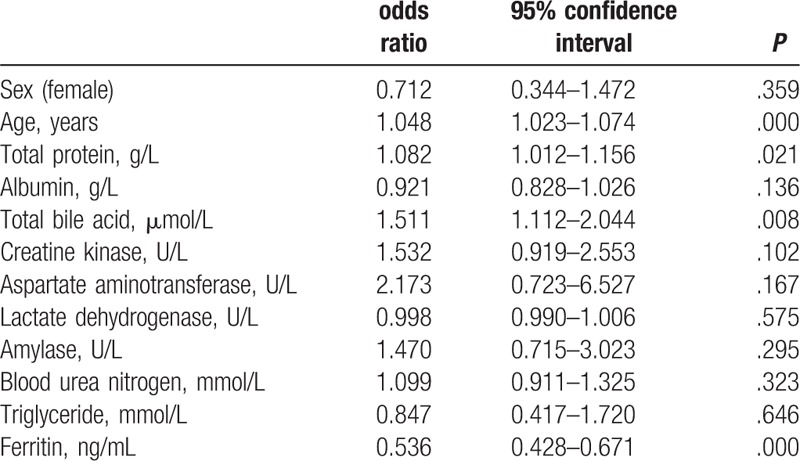
Logistic regression analysis of atrophic gastritis (defined as the pepsinogen I/II ratio < 3.0) in relation to potential variables (only partial variables listed).

## 4 Discussion

The present study explored the characteristics of the PG I/II ratio and their relationship with the nutritional and metabolic status of Chinese subjects. The prevalence of atrophic gastritis was 5.3% and increased significantly with age but did not differ between sexes. Age, total bile acid, total protein, and ferritin correlated independently with AG.

We adopted the criterion of a PG I/II ratio < 3 to screen for atrophic gastritis in a healthy population. Previous studies have shown that the ratio of PG I/II < 3 is closely related to atrophic gastritis, with 71% sensitivity, 86% specificity and 85% overall accuracy for atrophy detection.^[4]^ In total, 101 of 1896 subjects (5.3%) were found to have atrophic gastritis according to this criterion in our study. The prevalence of atrophic gastritis varied widely depending on race, region, diagnostic methods, and many other influencing factors, but the increasing trend with age, and the lack of a difference by sex were commonly seen, which is consistent with our study.

The first question addressed in this study involves the increasing tendency to develop atrophic gastritis with aging. It may result from the mucosal transition through different stages to chronic gastritis associated with *H pylori*, environmental factors, or autoimmunity against gastric glandular cells.

The second problem relates to protein metabolism. Protein digestion normally begins in the stomach with hydrolysis of protein molecules into proteose, peptone, and a small amount of amino acid by pepsin at acidic pH. In addition, the evacuation of the peptic digestion product and gastric acid from the stomach into the duodenum stimulates pancreatic secretion for further degradation. It is inferred that the lack of gastric pepsin and acid may impair protein digestion due to the decreased stimulation of the pancreas and bacterial overgrowth in the duodenum.^[14]^ Another possible explanation may be related to the decreased production of gastric ghrelin in patients with atrophic gastritis. Ghrelin is predominantly produced by the P/D1 cells of gastric oxyntic gland and can increase appetite and induce a positive energy homeostasis leading to weight gain.^[15]^ It has been found that plasma ghrelin level decreased with the progress of mucosal atrophy irrespective of Helicobacter pylori infection and correlated well with the serum level of the PG I and PG I/II ratio.^[16]^ The influence of decreased gastric pepsin, acid, and ghrelin may illustrate the relationship between the PG I/II ratio and serum albumin.

The third issue addressed is iron metabolism. Many investigations have demonstrated the important role of gastric hydrochloric and ascorbic acid in iron absorption.^[17,18]^ In the stomach, iron is reduced to ferrous iron by ascorbic acid and forms soluble chelates in acidic pH; then, in the duodenum, iron absorption is mediated by specific proteins under the regulation of hepcidin.^[19]^ Previous research has revealed that intragastric ascorbic acid is lower in patients with gastric atrophy and inversely related to pH and degree of atrophy.^[20,21]^ Decreased intragastric acidity and ascorbic acid due to atrophic gastritis may lead to decreased iron absorption or, even worse, to iron deficiency anemia. As for the role of hepcidin in the stomach, it is known as a regulatory hormone of iron homeostasis, predominantly expressed in the liver, and it plays an important part in iron absorption in duodenum and iron recycling by macrophages.^[19]^ Recent studies have found that hepcidin is also expressed in the fundus/corpus of the glandular stomach.^[22]^ One study found that serum prohepcidin, the precursor of hepcidin, was decreased in patients with atrophic gastritis, irrespective of *H pylori* infection, but no correlation was found between prohepcidin and iron metabolism indices;^[23]^ another study found that there was a statistically significant correlation between serum prohepcidin and ferritin.^[24]^ So far, there is still no investigation exploring the role that hepcidin plays in alterations of biochemistry and histopathology in patients with atrophic gastritis. Other studies also found that serum hepcidin was strongly associated with serum ferritin in a heathy population.^[25]^ However, as the results of the correlation between pro/hepcidin and iron metabolism parameters are so controversial, further studies are required to clarify the role of pro/hepcidin in atrophic gastritis.

An additional finding is that serum total bile acid is correlated with atrophic gastritis. This result may be explained by the speculation that patients with atrophic gastritis are predisposed to developing bacterial overgrowth due to hypochlorhydria or even achlorhydria. The possibility of using serum unconjugated bile acid as a diagnostic marker for small intestine bacterial overgrowth in humans has been explored.^[26,27]^ A study investigating the association of gallstone disease with serum total bile acid levels found that total bile acid levels were significantly higher in gallstone patients compared with controls and were also significantly higher in gallstone patients with small intestinal bacterial overgrowth compared with those patients without it.^[28]^ The overgrowth of anaerobes and some gram-positive aerobes in the duodenum could produce deconjugating and/or dehydroxylating enzymes, resulting in more free and secondary bile acids.^[29]^ The alteration of bile acids composition could interrupt the enterohepatic cycling of bile acid and favor more nonionic passive diffusion of bile acids into serum.^[30]^ Bacterial overgrowth may be silent and is not usually detected, and more research should be done to investigate the relation between low PG I/II ratio and serum total bile acid.

There are several limitations of our study. First, our study was observational and was designed to investigate the association of PG I/II and biochemical markers; in other words, we could not directly validate the effects of atrophic gastritis on biochemical markers. Second, we did not measure ghrelin, pro/hepcidin or microbiota in the gastrointestinal tract, which we assumed played a role in the relationship between the PG I/II ratio and these biomarkers. Further research is needed to explore the mechanism of the interrelationship between PG I/II ratio and biochemical markers.

In summary, the PG I/II ratio was independently correlated with age, total bile acid, total protein, and ferritin. The PG I/II ratio, usually as a marker of atrophic gastritis, was also associated with nutritional and metabolic status. Special attention should be paid to the metabolism of iron, protein, and bile acid in patients with a low PG I/II ratio.

## Author contributions

**Weiwei Su:** Concept/design, Data analysis/interpretation, Statistics, Drafting article.

**Bin Zhou:** Data collection.

**Guangming Qin:** Concept/design, Data analysis/interpretation, Statistics.

**Zhihao Chen:** Data collection, Statistics.

**Xiaoge Geng:** Data collection, Statistics.

**Xiaojun Chen:** Data collection, Statistics.

**Wensheng Pan:** Concept/design, Funding, Concept/design.

**Conceptualization:** Guangming Qin, Wensheng Pan.

**Data curation:** Weiwei Su, Guangming Qin, Zhihao Chen, Xiaoge Geng, Xiaojun Chen.

**Formal analysis:** Weiwei Su, Guangming Qin, Zhihao Chen.

**Funding acquisition:** Wensheng Pan.

**Investigation:** Bin Zhou, Zhihao Chen, Xiaoge Geng, Xiaojun Chen.

**Software:** Bin Zhou.

**Writing – original draft:** Weiwei Su.

**Writing – review & editing:** Wensheng Pan.
